# Cost-effectiveness analysis and prevention effects of ultra-orphan drugs for rare diseases: an *in silico* model applied to Cryopyrin Associated Periodic Syndromes (CAPS)

**DOI:** 10.1186/1546-0096-13-S1-P185

**Published:** 2015-09-28

**Authors:** L Trieste, O Della Casa Alberighi, G Turchetti, F Pierotti, L Accame, V Lorenzoni, J Frenkel, M Gattorno, P Quartier, A Martini

**Affiliations:** 1 Scuola Superiore Sant'Anna, Institute of Management, Pisa, Italy; 2 Istituto di Ricovero e Cura a Carattere Scientifico (IRCCS) Giannina Gaslini, Unità di Farmacologia Clinica e Sperimentazioni Cliniche, Direzione Scientifica, Genova, Italy; 3 University Medical Center Utrecht, Utrecht, Netherlands; 4 Istituto di Ricovero e Cura a Carattere Scientifico (IRCCS) Giannina Gaslini, Pediatria II, Reumatologia, Genova, Italy; 5 Assistance Publique-Hopitaux de Paris Hopital Necker,, Paediatric Immunology, Haematology and Rheumatology Unit, Paris, France

## Objectives

This three-year, international, multicentre, longitudinal, observational, cost-effectiveness study named RaDiCEA (RareDisease &Cost-EffectivenessAnalysis) will assess the economic evaluation (cost of illness - COI and cost-effectiveness analysis - CEA) of innovative therapies (i.e., anti IL-1 agents), quality of life (QoL) and effects of the prevention of otherwise irreversible central nervous system, eye, ear, kidney, and cartilage damages of different treatment strategies for cryopyrin-associated periodic syndromes (CAPS) of adults and children.

## Methods

A virtual time-cohort approach and a Markov model simulating health states corresponding to different CAPS severity will be developed to assess the cost-effectiveness of two different treatment strategies: i.e., either anti IL-1 agents or other than anti IL-1. Due to the lack of a CAPS-specific severity index/damage score, a linear combination of existing indexes and damage scores will be used to rank patient's health status with respect to damages involving specific organs and systems. Coefficients of the resulting function will be assigned following both a top-down (Delphi) and an interim-ex post-bottom-up approach (principal component analysis) considering covariances of all the variables adopted to describe the disease evolution or response to therapies. The model uses relevant economic measures to quantify resource utilization for patients' care in the National Health Systems' perspectives and a broader societal perspective to take into account direct nonmedical costs and indirect costs, in addition to direct costs. QoL will be evaluated using EQ-5D questionnaires. To assess how the model reacts to changes in singular and multiple disease parameters, univariate and probabilistic sensitivity analyses will be performed.

## Expected results

The RaDiCEA project will assess the long-term effectiveness of different potentially life-long treatment strategies and COI, while exploring the feasibility of a new CEA model to be generated from a rare disease (CAPS) observational study. The economic outcomes will be given as the number of years spent in each health state, the related yearly costs and QoL.

## Conclusions

The importance and novelty of the model is twofold: i) in its application, adopting the cost-effectiveness approach for assessing the impact of CAPS therapies, and ii) in the methods, extending the analyses of the impact of CAPS therapies in reducing the speed of disease progression.

